# SARS-CoV-2 infection activates a subset of intrinsic pathways to inhibit type I interferons *in vitro* and *in vivo*

**DOI:** 10.7150/ijms.56630

**Published:** 2021-04-28

**Authors:** Weisheng Luo, Lianzhou Huang, Xiaohui Wang, Yuying Ma, Ji Xiao, Xiaowei Song, Ping Liu, Yifei Wang, Yiliang Wang, Zhe Ren

**Affiliations:** 1 Guangzhou Jinan Biomedicine Research and Development Center, Institute of Biomedicine, College of Life Science and Technology, Jinan University, Guangzhou 510632, PR China.; 2 Key Laboratory of Virology of Guangzhou, Jinan University, Guangzhou 510632, PR China.

**Keywords:** SARS-CoV-2, innate antiviral responses, type I IFNs, immune evasion

## Abstract

SARS-CoV-2 infection poses a global challenge to human health. Upon viral infection, host cells initiate the innate antiviral response, which primarily involves type I interferons (I-IFNs), to enable rapid elimination of the invading virus. Previous studies revealed that SARS-CoV-2 infection limits the expression of I-IFNs *in vitro* and *in vivo*, but the underlying mechanism remains incompletely elucidated. In the present study, we performed data mining and longitudinal data analysis using SARS-CoV-2-infected normal human bronchial epithelial (NHBE) cells and ferrets, and the results confirmed the strong inhibitory effect of SARS-CoV-2 on the induction of I-IFNs. Moreover, we identified genes that are negatively correlated with *IFNB1* expression *in vitro* and* in vivo* based on Pearson correlation analysis. We found that SARS-CoV-2 activates numerous intrinsic pathways, such as the circadian rhythm, phosphatidylinositol signaling system, peroxisome, and TNF signaling pathways, to inhibit I-IFNs. These intrinsic inhibitory pathways jointly facilitate the successful immune evasion of SARS-CoV-2. Our study elucidates the underlying mechanism by which SARS-CoV-2 evades the host innate antiviral response *in vitro* and *in vivo*, providing theoretical evidence for targeting these immune evasion-associated pathways to combat SARS-CoV-2 infection.

## Introduction

SARS-CoV-2 is an emerging pathogen that poses a considerable threat to global human health, and a comprehensive understanding of the SARS-CoV-2-host interaction is urgently required 1. The host innate immune response is the first line of defense against viral infection 2-4. Upon the recognition of viral components by pattern recognition receptors (PRRs), host cells initiate a concerted innate antiviral response that primarily involves type I interferon (I-IFN) production and the subsequent upregulation of IFN-stimulated genes (ISGs), which encode a subset of antiviral proteins. Importantly, I-IFNs show impressive antiviral activity against SARS-CoV-2 4-6. However, several viral proteins have acquired the capability to evade the innate antiviral response via numerous mechanisms 7. In general, viral proteins suppress innate antiviral responses in two ways: by activating intrinsic immunosuppressive pathways or by directly targeting innate antiviral signaling components to inhibit signal transduction. For example, SARS-CoV, which is closely related to SARS-CoV-2, encodes numerous proteins that antagonize the induction of I-IFNs and the effector functions of downstream ISGs 8. The balance between the innate antiviral immune response and viral immune evasion is crucial to viral pathogenesis. Indeed, previous studies have reported that SARS-CoV-2 infection induces the expression of a very small subset of ISGs but virtually no I-IFNs 4, 9-13, suggesting the impressive immune evasion activity of SARS-CoV-2. Several studies have revealed that SARS-CoV-2 counteracts the host innate antiviral response 7, 13. A SARS-CoV-2 protein interaction network revealed that NSP13 of SARS-CoV-2 may interact with TBK1 14, although the function of this interaction in SARS-CoV-2 immune evasion remains unknown. Thus, the specific and precise mechanism by which SARS-CoV-2 antagonizes I-IFN signaling needs further exploration.

In the present study, we performed data mining and longitudinal data analysis of SARS-CoV-2-infected normal human bronchial epithelial (NHBE) cells and ferrets, and the results confirmed the strong ability of SARS-CoV-2 to inhibit the induction of I-IFNs *in vitro* and *in vivo*. We further analyzed genes that are negatively correlated with IFNB1 in SARS-CoV-2-infected NHBE cells and ferrets. Our study reveals the mechanism by which SARS-CoV-2 evades the host innate antiviral response and thus provides a potential therapeutic target for COVID-19.

## Results

### Analysis of data from SARS-CoV-2-infected NHBE cells demonstrated that SARS-CoV-2 infection inhibits the expression of I-IFNs in NHBE cells

**Figure 1:**
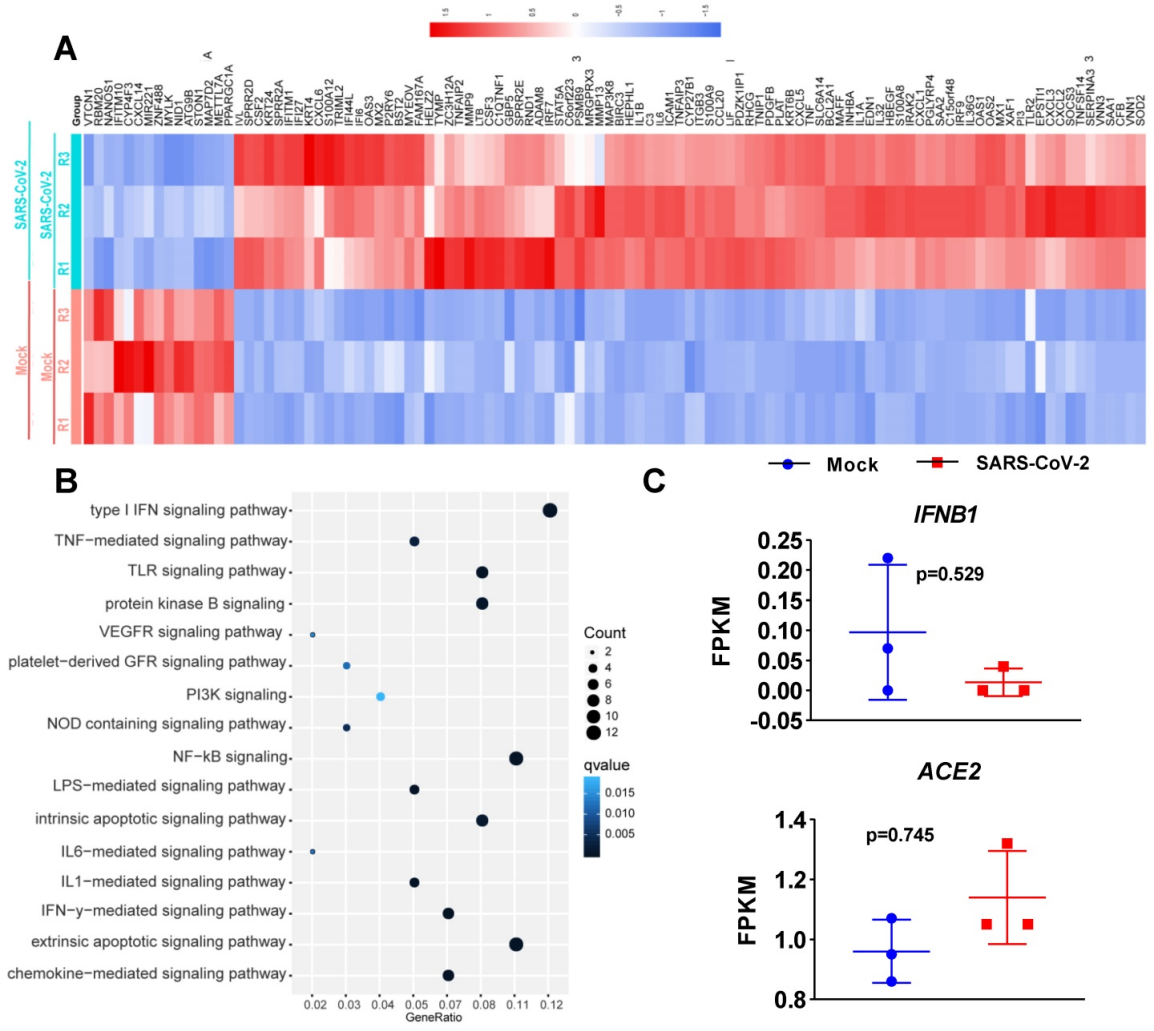
** Transcriptional response of primary human bronchial epithelial cells to SARS-CoV-2. A.** Heatmap of differentially expressed protein-coding genes in SARS-CoV-2-infected NHBE cells compared with the corresponding mock-infected cells. **B.** Dot plot visualization of enriched GO terms in SARS-CoV-2-infected NHBE cells. Gene enrichment analysis of the differentially expressed genes between SARS-CoV-2- and mock-infected NHBE cells was performed. The colors of the dots represent the q values for each enriched GO term, and their sizes represent the number of genes enriched in the corresponding signaling pathway. **C.** Levels of the innate antiviral factors *IFNB1*,* ACE2*,* IFI6*,* XAF1*,* MX1*,* MX2*, *IFITM1*,* IRF7*,* OAS1*,* OAS2*, *OAS3*,* IRF9*,* IFI27*,* ISG15*,* ISG20*,* OASL* and* CXCL10* in SARS-CoV-2- and mock-infected NHBE cells were determined as FPKM values. The p values were calculated with DESeq2 software.

**Figure 2:**
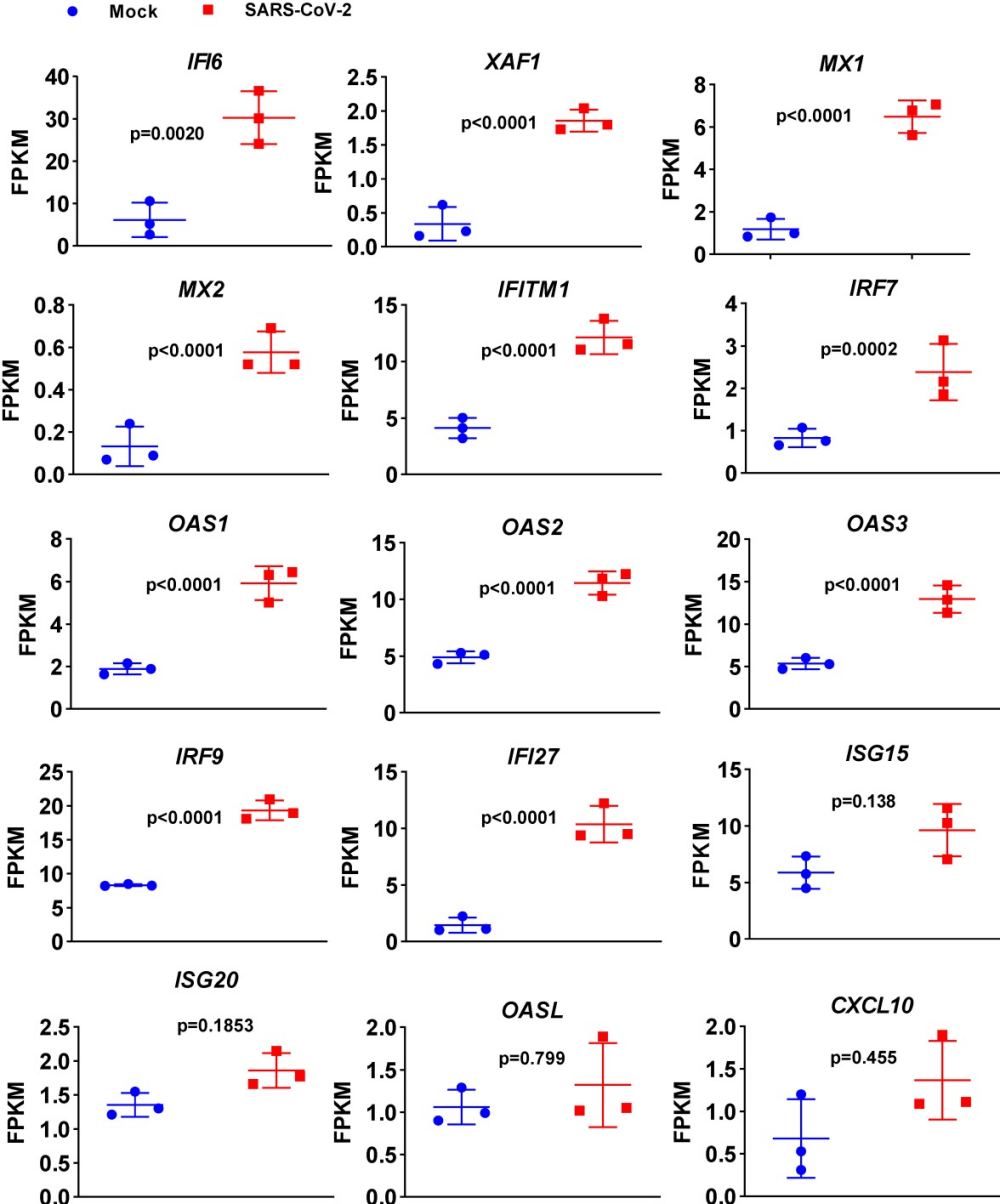
** Expression levels of a subset of ISGs in response to SARS-CoV-2 infection in primary human bronchial epithelial cells.** Levels of the innate antiviral factors* IFI6*, *XAF1*,* MX1*, *MX2*,* IFITM1*,* IRF7*,* OAS1*,* OAS2*, *OAS3*,* IRF9*, *IFI27*,* ISG15*, *ISG20*,* OASL* and* CXCL10* in SARS-CoV-2- and mock-infected NHBE cells are presented as FPKM values.

To determine the transcriptional response of NHBE cells to SARS-CoV-2, we performed differential expression analysis comparing SARS-CoV-2-infected cells to corresponding mock-infected cells based on previous transcriptome datasets 4. NHBE cells were infected with SARS-CoV-2 at an MOI of 2 for 24 h, and polyA RNA was subsequently enriched for subsequent RNA sequencing (RNA-seq) analysis 4. Our analysis of public transcriptome datasets identified 106 differentially expressed protein-coding genes (DEGs) in SARS-CoV-2-infected NHBE cells: 15 downregulated genes and 91 upregulated genes compared to their expression in control cells (**Figure 1A** and **Table S1**). To further understand changes in the expression profile of NHBE cells in response to SARS-CoV-2 infection, gene enrichment analysis of these DEGs was performed. The analysis revealed strong enrichment of these genes in chemotactic and inflammatory response signaling (**Figure 1B**). Specifically, upon SARS-CoV-2 infection, NHBE cells mount a chemokine-mediated signaling response characterized by the induction of *CCL20*,* CXCL6*,* CXCL1*,* CXCL5*,* CXCL3*,* CXCL2*, and *EDN1* (**Figure 1B and Table S2**). Consistent with previous publications 15-17, SARS-CoV-2 infection was also found to activate interleukin-6 (IL-6)-mediated signaling, which contributes to cytokine release syndrome in severe COVID-19, with representative genes of this pathway including *SOCS3* and *IL6* (**Figure 1B and Table S2**). SARS-CoV-2 infection also activated genes downstream of IκB kinase/NF-κB signaling, including *ZC3H12A*,* S100A12*,* BIRC3*,* BST2*,* IL1B*,* IL36G*,* IRAK2*,* TLR2*,* TNIP1*,* TNF*, and *TNFAIP3* (**Figure 1B and Table S2**). However, the infection of NHBE cells with SARS-CoV-2 failed to induce the expression of I-IFNs, including* IFNB1* and a series of IFN genes, as supported by the quantitative results for all protein-coding transcripts (**Figure 1C** and **Table S1**). Notably, although a significant increase in *IFNB1* was not observed in SARS-CoV-2-infected NHBE cells, ISGs were induced by SARS-CoV-2 infection (**Figure 2**). The ISGs induced by SARS-CoV-2 infection included *IFI6*,* XAF1*,* IFITM1*,* IRF7*, *OAS1*, *OAS3*,* OAS2*,* IRF9*,* IFI27*,* XAF1*,* BST2*,* MX2*, and* MX1* (**Figure 2**). In contrast, the expression of several other ISGs, primarily *ISG15*,* ISG20*,* OASL*, and *CXCL10*, was not induced by SARS-CoV-2 (**Figure 2**). Notably, the expression of ACE2, a receptor for SARS-CoV-2 entry and recently identified ISG 18, was also slightly higher in SARS-CoV-2-infected NHBE cells than in control cells (**Figure 2**). These results indicate that SARS-CoV-2 exhibits the strong ability to inhibit *IFNB1* expression to enable its successful immune escape.

### Analysis of longitudinal data from SARS-CoV-2-infected ferrets demonstrated transient induction of IFNB1 by SARS-CoV-2 infection *in vivo*

**Figure 3:**
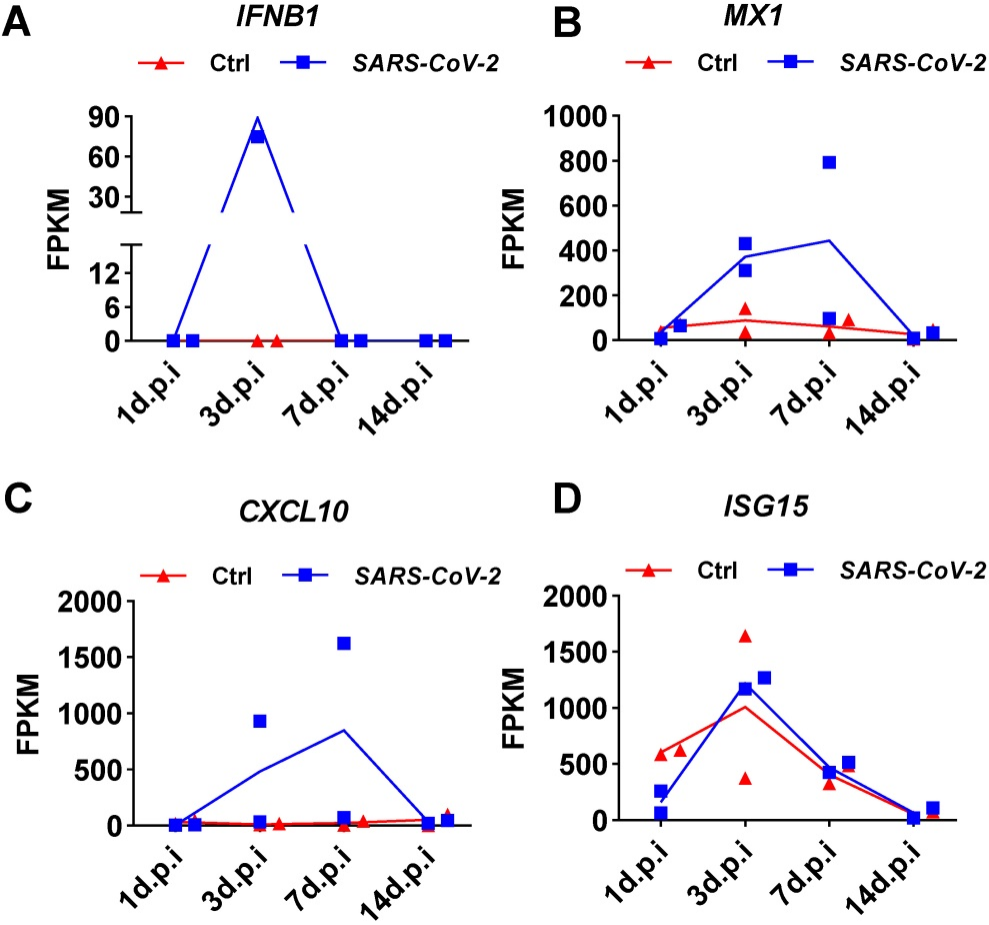
** Expression changes in IFNB1 and several ISGs in nasal washes samples collected from SARS-CoV-2- and mock-infected control ferrets. A-D.** Results of transcriptome analysis indicating the levels of IFNB1 (A) and several ISGs (B, C, and D) in nasal washes samples collected from SARS-CoV-2- and mock-infected control ferrets at indicated time points post-infection. The FPKM values of these genes are presented and reflect their expression levels.

To determine whether the weak induction of *IFNB1* by SARS-CoV-2 observed in NHBE cells was an artifact of the cell culture conditions, we analyzed public transcriptome data obtained from SARS-CoV-2-infected ferrets. Naive ferrets were intranasally infected with 5 × 10^4^ PFU of the SARS-CoV-2 isolate USA-WA1/2020 4. Small pellets consisting of upper respiratory tract cells isolated from nasal washes were subjected to transcriptome analysis. Transient induction of *IFNB1* by SARS-CoV-2 infection in ferrets over time was shown by the quantitative results presented as the corresponding fragments per kilobase million (FPKM) values (**Figure 3**). Specifically, on day 1 after infection, no SARS-CoV-2-mediated induction of *IFNB1* was observed (**Figure 3A**). Significant *IFNB1* upregulation was observed at 3 days post-infection (**Figure 3A**). However, the expression of *IFNB1* quickly decreased to the baseline level at 7 days post-infection (**Figure 3A**). Inconsistent with the observed trend in the *IFNB1* level, persistent upregulation of numerous ISGs, including *CXCL10*,* MX1*, and *ISG15*, was observed in response to SARS-CoV-2 infection from 3 to 7 days post-infection (**Figure 3B, 3C, and 3D**).

**Figure 4:**
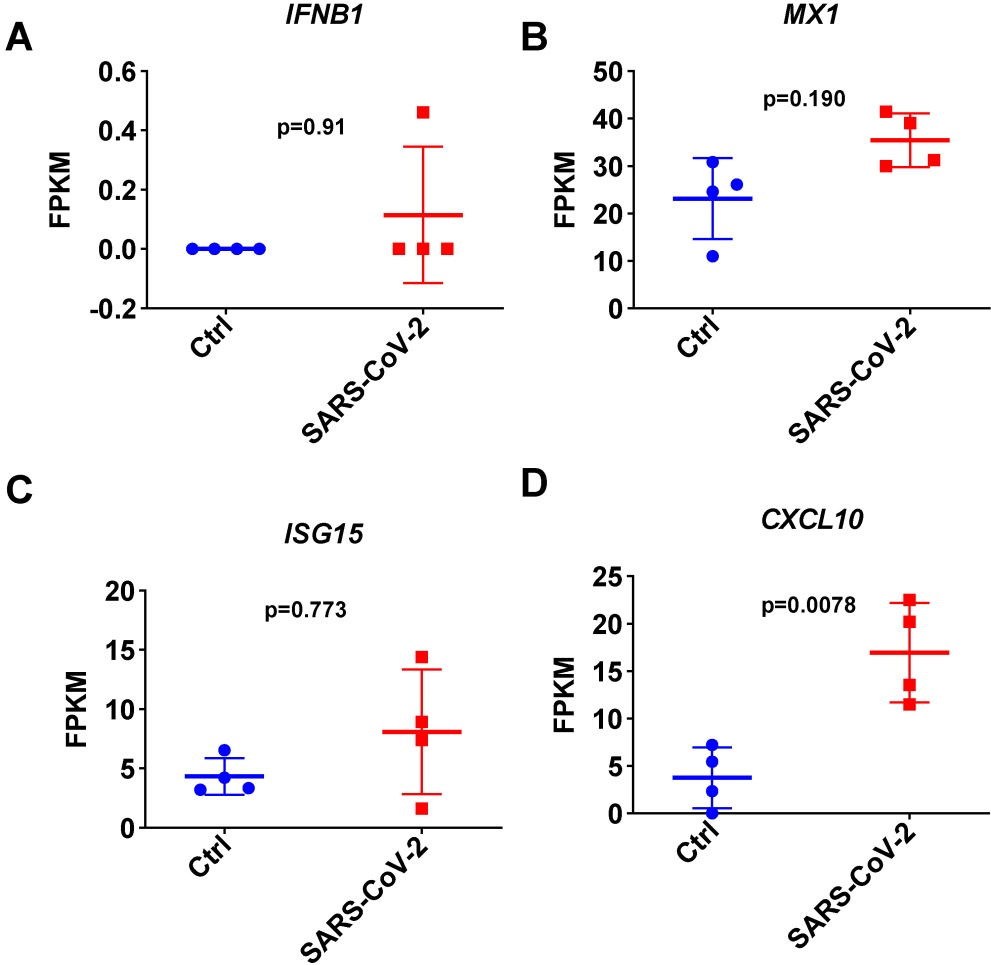
** Expression changes in IFNB1 and several ISGs in respiratory tract samples collected from SARS-CoV-2- and mock-infected control ferrets. A-D.** Results of transcriptome analysis indicating the levels of IFNB1 (A) and several ISGs (B, C, and D) in respiratory tract samples from SARS-CoV-2- and mock-infected control ferrets on day 3 post-infection. The FPKM values of these genes are presented and reflect their expression levels.

To further investigate the I-IFN response to SARS-CoV-2 in detail, we next focused on analyzing the respiratory tract in SARS-CoV-2-infected ferrets infected in parallel on day 3 using public transcriptome data 4. Consistent with the results observed with the small cell pellets, the quantitative results of transcriptome data analysis indicated that SARS-CoV-2 infection inhibited the expression of *IFNB1* in the respiratory tracts of SARS-CoV-2-infected ferrets on day 3 (**Figure 4A**). Although SARS-CoV-2 infection induced significant *CXCL10* upregulation, most ISGs were not significantly upregulated in the respiratory tract in SARS-CoV-2-infected ferrets (**Figure 4B, 4C, and 4D**). These results indicated that SARS-CoV-2 infection induces the transient expression of *IFNB1* but causes an insufficient response to I-IFNs in the respiratory tract. The upregulation of ISGs observed in the nasal wash samples may be attributable to the small size of the upper respiratory tract cell pellets.

### Analysis of SARS-CoV-2-induced genes whose expression is negatively correlated with IFNB1* in vitro* and *in vivo*

**Figure 5:**
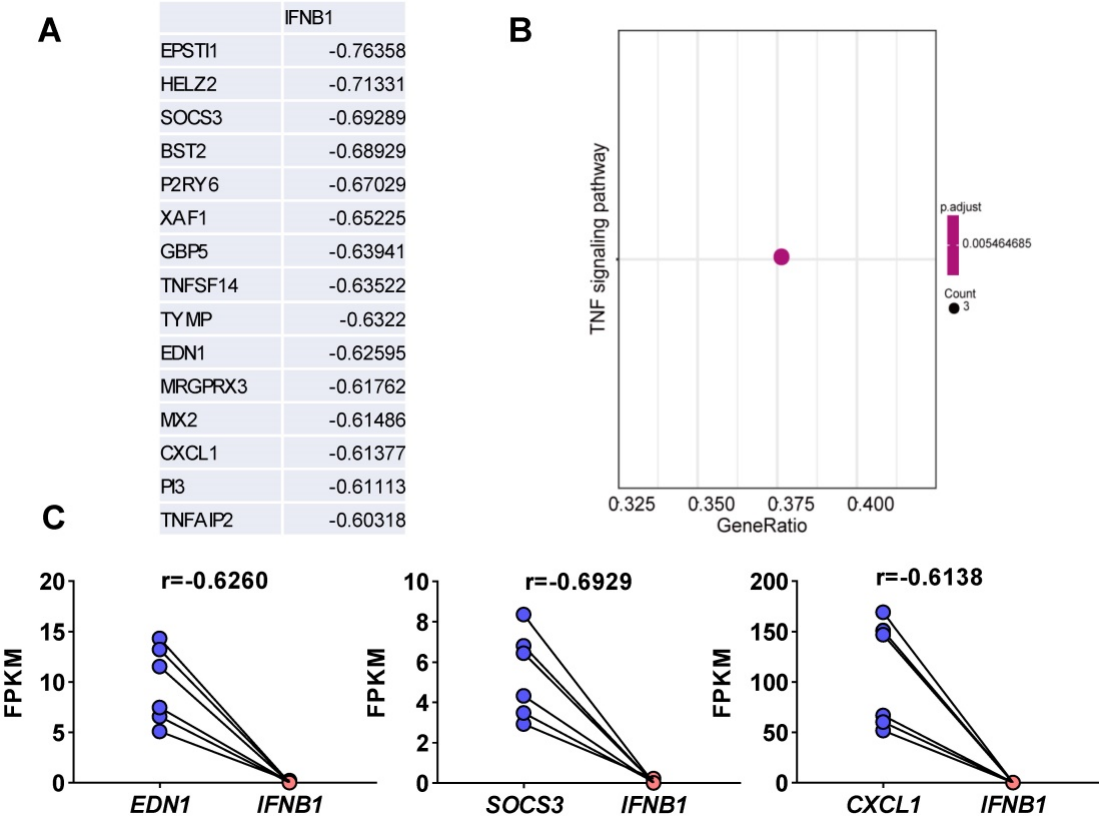
** Elucidation of SARS-CoV-2-induced genes whose expression was negatively correlated with *IFNB1* expression *in vitro.* A.** Pearson correlation coefficient-based analysis of genes whose expression was negatively correlated with IFNB1 expression. The expression of 15 genes was negatively correlated with that of *IFNB1*: *EPSTI1*,* HELZ2*,* SOCS3*, *BST2*,* P2RY6*, *XAF1*,* GBP5*,* TNFSF14*,* TYMP*,* EDN1*, *MRGPRX3*,* MX2*,* CXCL1*, *PI3*, and* TNFAIP2*. **B.** The results of KEGG analysis showed that the correlated genes were enriched in the TNF signaling pathway (*SOCS3*,* CXCL1*, and* EDN1*; adjusted p value=0.005464685 and gene count=3). **C.** The levels of genes enriched in the TNF signaling pathway upon SARS-CoV-2 infection in NHBEs. The FPKM values of the indicated genes were calculated and reflect the expression levels of these genes. The correlation coefficients (r) between *IFNB1* and the corresponding genes are also indicated.

**Figure 6:**
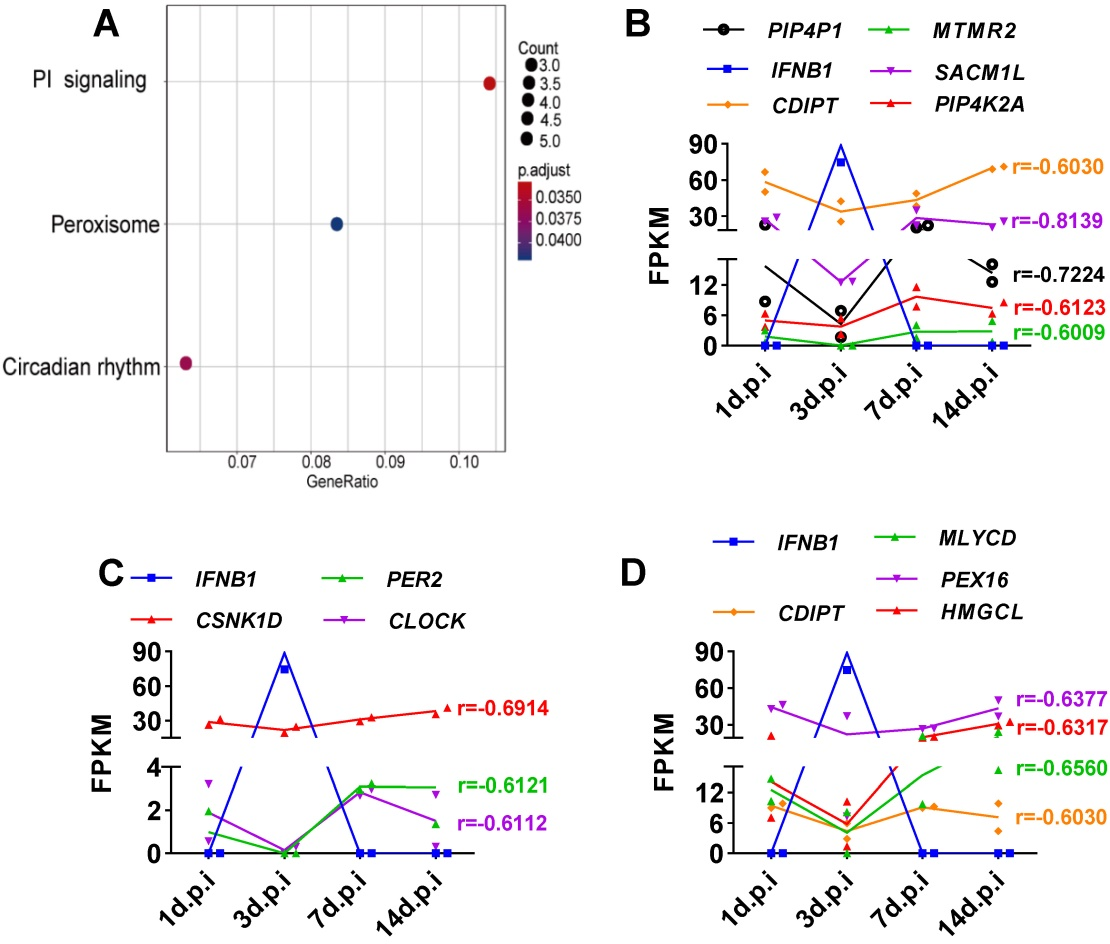
** Elucidation of SARS-CoV-2-induced genes whose expression was negatively correlated with IFNB1 expression in ferrets with SARS-CoV-2 infection. A.** Based on the obtained Pearson correlation coefficients, the results of KEGG analysis revealed that genes that are negatively correlated with *IFNB1* were primarily enriched in the phosphatidylinositol (PI) signaling system, circadian rhythm, and peroxisome pathways. **B-D.** Longitudinal changes in genes enriched in the signaling pathways shown in (A), the PI signaling system(B), circadian rhythm (C), and peroxisome (D) pathways, in SARS-CoV-2-infected ferrets. The levels of these genes are presented as FPKM values. The correlation coefficients (r) between IFNB1 and the corresponding genes are also indicated.

Viruses usually encode numerous type I IFN antagonists to ensure successful evasion of host innate immunity 7, 14. These antagonists suppress the expression of I-IFNs by activating intrinsic pathways, restricting I-IFN induction, or directly manipulating the crucial factors that activate these I-IFN pathways. To identify the pathway by which SARS-CoV-2-mediated I-IFN inhibition is activated, we analyzed SARS-CoV-2-induced genes that were negatively associated with *IFNB1 in vitro* and *in vivo* based on Pearson correlation analysis. We identified 15 genes whose expression was negatively correlated with* IFNB1* expression: *EPSTI1*,* HELZ2*,* SOCS3*,* BST2*,* P2RY6*,* XAF1*,* GBP5*,* TNFSF14*,* TYMP*,* EDN1*,* MRGPRX3*,* MX2*,* CXCL1*,* PI3*, and *TNFAIP2* (**Figure 5A and Table S3**). The results of KEGG analysis suggested that a number of these correlated genes were enriched in the TNF signaling pathway (**Figure 5B**); these genes included *SOCS3*,* CXCL1*, and *EDN1* (**Figure 5C**). Moreover, we identified genes that were negatively correlated with *IFNB1* in our longitudinal study of SARS-CoV-2-infected ferrets via Pearson correlation-based analysis (**Table S4**). The results of KEGG analysis revealed that the genes that were negatively correlated with *IFNB1* in SARS-CoV-2-infected ferrets were primarily enriched in the following pathways: the phosphatidylinositol signaling system (*PIP4P1*,* MTMR2*,* SACM1L*,* CDIPT*, and *PIP4K2A*), the circadian rhythm (*PER2*,* CSNK1D*, and *CLOCK*), and peroxisomes (*CDIPT*, M*LYCD*,* PEX16*, and *HMGCL*) (**Figure 6**).

## Discussion

Numerous studies have reported that SARS-CoV-2 infection induces a weakened innate antiviral defense response characterized by a low level of I-IFNs and a strong proinflammatory response 4, 7, 15, 18. I-IFNs are the primary initiators that activate the expression of antiviral genes. Indeed, patients with life-threatening COVID-19 show an inherited defect in the innate antiviral response 19, 20, further implying the importance of I-IFNs in combating SARS-CoV-2 infection. However, the underlying mechanisms by which type I IFNs are inhibited by SARS-CoV-2 remain largely unknown. In general, viruses can produce numerous proteins that target crucial factors involved in I-IFN signaling or activate intrinsic pathways to inhibit I-IFN signaling and achieve immune evasion 8, 21-23. The activation of intrinsic pathways that inhibit I-IFNs is a crucial negative feedback mechanism that maintains host immune hemostasis to avoid damage due to the excessive production of I-IFNs 22. In the present study, we focused on elucidating the intrinsic inhibitory pathways activated by SARS-CoV-2 infection* in vitro* and *in vivo*.

First, we confirmed that SARS-CoV-2 infection inhibited the expression of *IFNB1 in vitro* and *in vivo* by analyzing published *in vitro* and *in vivo* transcriptome data, and our findings were consistent with those of previous studies showing that SARS-CoV-2 infection triggers decreased I-IFN production 4, 9-13. Specifically, SARS-CoV-2 infection induced limited IFNB1 expression. In contrast, SARS-CoV-2 infection inhibited the expression of *IFNB1* in NHBE cells. Moreover, although the expression of most ISGs could not be induced by SARS-CoV-2 infection, the expression of several ISGs was induced. Taken together, these findings indicate that SARS-CoV-2 infection induced a weakened innate antiviral response *in vitro*. Similar to the results observed *in vitro*, SARS-CoV-2-infected ferrets showed the transient induction of *IFNB1*, as demonstrated by transcriptome analysis of the respiratory tract and nasal wash samples. Given the suppressed innate antiviral response observed in the context of SARS-CoV-2 infection, we speculated that SARS-CoV-2 exhibits strong immune evasion activity. Thus, we performed Pearson correlation analysis to analyze genes whose expression was negatively correlated with *IFNB1* expression in the context of SARS-CoV-2 infection *in vitro* and* in vivo*. The *in vitro* correlation analysis results suggested that the enrichment of primarily the SOCS3, EDN1, and CXCL1 genes was negatively correlated with IFNB1. SOCS3, a component of the TNF signaling pathway, is a factor known to inhibit overactive I-IFN signaling 22. Given that TNF activates an IRF1-dependent autocrine loop, leading to sustained expression of STAT1-dependent I-IFN response genes 24, the activation of TNF signaling may explain the induction of ISGs but not *IFNB1*. Indeed, accumulating evidence indicates that anti-TNF therapy shows impressive efficacy against COVID-19 and should accordingly receive priority in COVID-19 treatment trials 25. In contrast, no studies have addressed the roles of *EDN1* and *CXCL1* in suppressing I-IFN signaling. The *in vivo* correlation analysis revealed that SARS-CoV-2 infection significantly activates the circadian rhythm, phosphatidylinositol signaling, and peroxisome signaling pathways, all of which may jointly contribute to suppressing the innate antiviral response to SARS-CoV-2 *in vivo*. Indeed, the disruption of circadian rhythms has been suggested to lead to the impairment of innate immunity 26-28. In particular, mice lacking circadian rhythms showed robust activation of the IFN pathway 27, and the circadian clock has been shown to regulate cellular susceptibility to the herpes and influenza viruses 29. A recent study revealed that systemic Bmal1-knockout (KO) mice had an exacerbated imiquimod-induced ISG response in the skin and isolated epidermis, suggesting an involvement of the circadian clock in the innate antiviral response 27. Indeed, the importance of the circadian clock in COVID-19 management has been demonstrated, although the associated mechanism remains obscure 30. Peroxisomes are also signaling platforms for antiviral innate immunity 31, implying that they may be involved in limiting the innate antiviral response to SARS-CoV-2. However, a limitation of our present study is that we performed only transcriptome analysis, and the findings need further experimental confirmation. Despite this limitation, our study provides novel insight into the mechanisms underlying the immune evasion of SARS-CoV-2. An inhibitor targeting inhibition of the intrinsic pathway of I-IFN activated by SARS-CoV-2 is expected to be an ideal therapeutic target for COVID-19 by initiating intrinsic innate antiviral immunity.

## Materials and Methods

### Acquisition and analysis of high-throughput RNA-seq data

The raw high-throughput RNA-seq data analyzed in the present study were obtained from GEO (GSE147507) and published in a previous study 4. The method used to analyze the raw data in the present study was similar to that described in our previous study with minor modifications 32. FastQC software with the default parameters was used to filter the low-quality reads to obtain clean data of high quality. The filtered RNA-seq data were then mapped to the genome of the corresponding species using HISAT2 v2.0.4 33. The mapped reads of each sample were assembled with StringTie (v1.3.1) via a reference-based approach 34. StringTie uses a novel network flow algorithm as well as an optional *de novo* assembly step to assemble and quantitate full-length transcripts representing multiple splice variants for each gene locus 34. The FPKM value is an indicator of gene expression. DEGs were identified using the R package DESeq2 v4.0 35. An adjusted p value < 0.05 (according to the Benjamini-Hochberg procedure) 36 and an absolute fold change > 2 were considered to indicate significantly differential gene expression.

### Pearson correlation analysis

Correlations between *IFNB1* and other genes were analyzed by Pearson correlation analysis 37. In brief, we calculated the correlation coefficients (r, Pearson correlation coefficient; p value ≤ 0.05) between IFNB1 and other genes and used the criterion |r|≥0.6 to identify the correlated genes. Pearson correlation analysis was conducted using the Python package scipy 38.

### Pathway enrichment analysis

Genes usually interact to take part in specific biological functions. Pathway-based analysis facilitates further understanding of the biological functions of genes, and KEGG is a major public pathway-related database 39, 40. In the present study, KEGG enrichment analysis was performed with the R package clusterProfiler v3.0.4 41. Pathway enrichment analysis identified metabolic pathways and signal transduction pathways significantly enriched in the DEGs compared to the whole-genome background using the formula presented below. Specifically, N is the number of all genes with KEGG annotations, n is the number of DEGs among the N genes, M is the number of all genes annotated to specific pathways, and m is the number of DEGs among the M genes. The calculated p value was subjected to FDR correction, with FDR ≤ 0.05 as the threshold. Pathways meeting this condition were defined as pathways significantly enriched in DEGs.


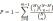


## Supplementary Material

Supplementary tables.
